# Conceptual preferences can be transmitted via selective social information use between competing wild bird species

**DOI:** 10.1098/rsos.220292

**Published:** 2022-06-15

**Authors:** Jukka T. Forsman, Sami M. Kivelä, Jere Tolvanen, Olli J. Loukola

**Affiliations:** Department of Ecology and Genetics, University of Oulu, PO Box 3000, 90014, Finland

**Keywords:** concept learning, social learning, interspecific social information use, great tit, pied flycatcher

## Abstract

Concept learning is considered a high-level adaptive ability. Thus far, it has been studied in laboratory via asocial trial and error learning. Yet, social information use is common among animals but it remains unknown whether concept learning by observing others occurs. We tested whether pied flycatchers (*Ficedula hypoleuca*) form conceptual relationships from the apparent choices of nest-site characteristics (geometric symbol attached to the nest-box) of great tits (*Parus major*). Each wild flycatcher female (*n* = 124) observed one tit pair that exhibited an apparent preference for either a large or a small symbol and was then allowed to choose between two nest-boxes with a large and a small symbol, but the symbol shape was different to that on the tit nest. Older flycatcher females were more likely to copy the symbol size preference of tits than yearling flycatcher females when there was a high number of visible eggs or a few partially visible eggs in the tit nest. However, this depended on the phenotype, copying switched to rejection as a function of increasing body size. Possibly the quality of and overlap in resource use with the tits affected flycatchers' decisions. Hence, our results suggest that conceptual preferences can be horizontally transmitted across coexisting animals, which may increase the performance of individuals that use concept learning abilities in their decision-making.

## 1. Introduction

Optimal behaviour requires information about current and future conditions. An important way to perceive the world and adapt one's own behaviour is a capability to form relative associations, such as larger–smaller, better–worse and same–different, between stimuli. Such an ability—concept learning—is considered higher order learning and adaptive because it enables individuals to extrapolate learned associations into different contexts, thereby reducing the time used in learning and decision-making [1–3]. Often thought to be solely a human trait and forming the foundations of human cognition and culture [1], recent studies have shown that concept learning exists widely in the animal kingdom, in non-primate mammals [1,4,5], birds [6,7] (see [3] for a review) and insects [8–10] (see [11] for a review). Our current knowledge of concept learning comes from laboratory-reared animals that have been trained to solve tasks in isolation via asocial trial and error learning using either repeated rewards or punishment (or both) (but see [12]). While these studies laid the foundation for the study of concept learning, they miss an important dimension of learning: how social learning in which the behaviour and choices of other coexisting individuals [13–16], including heterospecifics [17,18], is used to guide one's own behaviour. Asocial and social learning most likely depend on the same learning mechanisms [19], but it is unknown whether concept learning through social learning exists. If concept learning can take place by observing others, it would substantially expand our understanding of how animals perceive the world and adjust their own behaviour.

Cavity nesting resident great tits (*Parus major*) and migratory flycatchers (*Ficedula* spp.) form an ideal system to study various types of social information use and learning in the wild. Great tits and flycatchers have overlapping resource needs, and they are putative competitors [20,21], which is predicted to increase the probability of interspecific information use [17]. Indeed, flycatchers use tits as a source of information when making offspring investment decisions [21,22] and seek the vicinity of tits because they have a higher fitness there [23]. Moreover, using an apparent novel niche experiment (ANNE) match-to-sample task, we have shown that flycatchers not only copy the apparent choices of tits [24] but also selectively either copy or reject tit preferences, depending on the perceivable fitness correlate (number of eggs) of the tits [25–27]. ANNE uses abstract symbols, such as triangles and circles on the nest-boxes, that reflect the niche dimensions of the tits and especially portray the apparent preferences of nest-site characteristics of tits.

**Figure 1.**
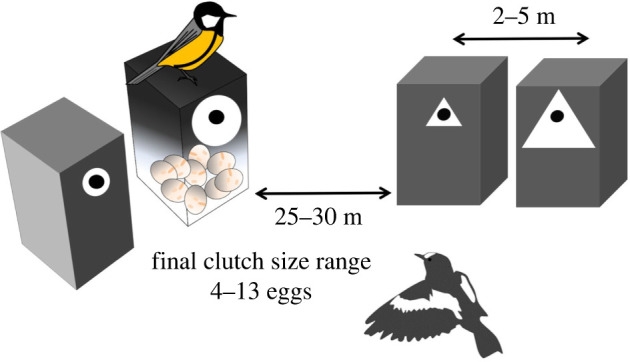
The experimental set-up. This design was intended to simulate a situation for pied flycatchers that great tits had apparently preferred either of the size of the symbol (large circle in this example) and flycatchers were then given a choice to either copy or reject the size concept preference of great tits but the symbol shape on available nest-boxes (triangle in this example) was different from those on the great tit nest-boxes. The order of the large/small symbol in each nest-box pair was randomized as well as the symbol shape (circle/triangle) attached to great tit nest-boxes. Replicate set-ups were set at least 1 km apart to ensure independence.

Here, we use a field experiment to test whether concept learning (or in a broader context, conceptual social information use) exists in an interspecific context and to determine which individual characteristics of the demonstrator and observer species affect the manifestation of learning. We used wild resident great tits (the demonstrator species) and migratory pied flycatchers (the observer species) as study organisms and the selection of nest-site characteristics as the behavioural trait. We modified our ANNE design and tested whether flycatchers use the relative size difference of abstract symbols on tit nests as a cue in their own nest-site choice (figure 1). This brings the larger–smaller concept into the experiment. Each flycatcher female observed one demonstrator tit pair that exhibited an apparent novel preference for either large or small symbols (triangle or circle) and was then allowed to choose between two nest-boxes with large or small symbols that were different from that on the demonstrator tit nest (figure 1).

Based on our previous study manipulating the clutch size of the tits [27], we expect the demonstrator tit's clutch size and its visibility (but not the phenotype of the tits) to affect the flycatcher's probability of copying or rejecting the tit's relative size concept preference. Flycatchers visit tit nests upon arrival from migration [28,29] and plausibly then can perceive the clutch size of tits. Tit individuals with good breeding performance plausibly make better choices [30], meaning that copying *any* of their preferences (such as tit's symbol size preference here) or choices (e.g. nest-site choices, foraging decisions and anti-predator strategies) is potentially adaptive [14,31,32]. Conversely, choices of poorly performing individuals are plausibly poor and thus should be rejected to avoid potentially poor outcomes. Hence, we predict that flycatcher symbol size preferences should more often match with tits having a high number of visible eggs, while preferences of tits with a low number of visible eggs should be actively rejected [26,27]. The age of the flycatcher females may also affect social information use [27] and old flycatcher females are predicted to have a higher probability to copy apparent tit choices than females breeding for the first time. There is also a genetic component in the preference of nest-site characteristic [33,34]. Nest-site is an important element of ecological niche in birds and can be under divergent selection among species because of the increasing risk of nest-predation with increasing similarity of nest-site characteristics [35]. Finally, we hypothesize that the phenotype of flycatcher females affects the interspecific information use because it affects resource use overlap and thus the intensity of competition with the demonstrator species [17]. Body size is an important trait affecting resource use [36], so we expect that the body size of a flycatcher female affects its copying or rejection decision. Flycatchers are smaller than great tits (average body masses *ca* 14–15 g versus 20–21 g), and we expect that increasing body size of flycatchers increases the probability of rejecting apparent size choice of tits to avoid the possible costs of overlap in resource use [36] and trade-offs in the value of information and costs of competition [17].

## 2. Methods

### 2.1. Set-up of the experiment

The experiment was conducted in mixed and coniferous forests near the city of Oulu, in northern Finland, between 2012 and 2014. Nest-boxes were situated along small roads and great tits were allowed to settle and breed freely. The basic sampling unit in the study was a set of two pairs of closely situated nest-boxes inhabited by a pair of great tits and a pair of pied flycatchers (figure 1). Prior to the arrival of flycatchers, we randomized one of the symbol shape (triangle/circle)–symbol size (large/small) associations and attached a white plastic symbol around the entrance hole of an active great tit nest. An empty nest-box was then erected near (3–6 m) the great tit's nest-box on a tree of the same species, at the same height and facing same direction as the occupied box, and the same symbol shape but opposite symbol size compared to that on the tit nest-box was attached on the empty box (figure 1). Two empty boxes, entrance holes facing towards the tit box pair, were erected 20–25 m from the great tit's nest-box and 3–6 m apart from each other, with both symbol sizes, but the opposite symbol shape compared to that on the great tit's nest-box randomized on the boxes. These two vacant nest-boxes were meant for flycatchers to choose between. We put one litre of sawdust into both vacant nest-boxes because flycatchers highly prefer it as a nest platform over empty nest-boxes or great tit nests [37]. This reduces the risk that pied flycatchers take over the great tit nest or choose the empty nest-box near the great tit nest. These set-ups were situated 1 km apart to minimize the risk that flycatchers perceive more than one symbol size association apparently preferred by great tits.

This design was intended to inform arriving flycatchers that the resident great tit pair had apparently preferred one of the sizes of the symbol. Flycatchers were then given a choice to either copy or reject the size concept preference of tits with symbols whose shape was different from that on tit nest. In this way, the only cues provided by the tits that the flycatchers could use were the nesting success (clutch size) and its association with the symbol size (large/small). The sizes of the symbols were small and large triangle: 17.7 and 83.6 cm^2^, respectively, and small and large circle: 19.5 and 40.5 cm^2^, respectively. The size differences between small triangles and small circles as well as large triangles and large circles were about 10% and 50%, respectively, and were chosen to avoid the possibility that flycatchers would use the surface area of the small and large symbols in making the choice and not the relative size difference. To avoid the possibility that the previous year's symbol shape and size preferences of tits affect current choices, the study area was changed annually. Great tits and pied flycatchers were breeding in natural cavities in the study area in low numbers but there were no nest-boxes other than ours in the area.

### 2.2. Measurements

We visited the nests at least every second day and determined the choice of each flycatcher female upon the appearance of nest material in the nest-box. During egg-laying, great tits usually cover eggs totally or partially but sometimes leave them totally exposed. We therefore monitored how many eggs were visible and the clutch size in great tit nests on the day of flycatcher choice. Once the flycatcher had made a choice, all symbols and the two empty nest-boxes were removed. Flycatcher females were captured during incubation and their age was estimated and their tarsus length was measured. Age of the females was estimated from tail and wing feathers as young (born in the previous year and therefore breeding for the first time) or adult (at least 2 years old and probably has bred earlier) [38]. All birds were handled and measured by J.T.F. under his ringing licence (2975) and following the Finnish legislature.

### 2.3. Statistical analyses

Usually, most tits have initiated egg-laying prior to the arrival of flycatchers [29]. However, in our data, 21 flycatchers out of 124 made a choice and settled before great tits had laid their first egg. We therefore analysed the data in two parts: (i) data including choices made during egg-laying or incubation of great tits and (ii) data including all choices irrespective of the state of great tit reproductive cycle. Great tit nests where egg-laying was not initiated before flycatcher choice mostly consisted of undeveloped moss nests or nearly/fully finished nests with hair cover but where egg-laying was not yet initiated, or were deserted nests and did not result in a nesting attempt (but at the moment of flycatcher choice it was not known whether the great tit territory was active or not). This division is biologically meaningful because our previous studies have demonstrated that the perceivable clutch size of the tits provides important information for flycatchers and strongly affects their choices [25–27,39]. The possibility to perceive eggs in great tit nests also confirms that the great tit territory is active because tits often start nest-building in several cavities, one of which is chosen. The level of testosterone also decreases from nest-building to egg-laying state [40], which plausibly reflects to the behaviour of great tits. In the main text, we present the results of the data including choices made during egg-laying or incubation of great tits, while the results of the whole data are shown in the electronic supplementary material.

In both datasets, we tested how the phenotype of the nest of the demonstrator tit and the phenotype of the flycatcher female explain flycatcher choices. We used generalized linear models (R function *‘*glm’, binomial error distribution and logistic link function) to determine if tit clutch size (6.1 ± 0.28 s.e. eggs), the proportion of visible eggs on the day of flycatcher choice (0.56 ± 0.046), the phenotype of the flycatcher female in terms of morphology (length of tarsus; 19 ± 0.047 mm) and age (adult/born in the previous year; 59 adults and 44 yearlings) explain the symbol size choice of the flycatcher female (binary variable: matching (44 times; coded as 1) versus opposing (59 times; coded as 0) symbol size choice than that on the tit's nest). The full model included all main effects, the three-way interaction among tit clutch size at the time of choice, the proportion of visible clutch size and flycatcher female age, as well as all two-way interactions among these three variables. Visual evaluation of residual plots indicated that the full model fitted the data well, and residual deviance (114 on 97 degrees of freedom) did not indicate overdispersion.

All analyses were conducted using R (v. 4.1.2) [41]. We derived all meaningful sub-models of the full model with the function *‘*dredge’ [42], ranked the models using Akaike's information criterion adjusted for small sample sizes (AICc) [43] and derived the 95% confidence set of models (i.e. the set of models over which the cumulative sum of Akaike weights is less than or equal to 0.95) for further consideration. We further removed all models that were more complex extensions of the model with the lowest AICc value from the model set (suggested by [43,44] and all models that were greater than 6 AICc units from the best model (suggested by [44]). We used ΔAICc, Akaike weights of the models and their evidence ratios to determine if there was a single superior model explaining the data or a set of nearly equal models. If there was no single best model (ΔAICc less than 2 and evidence ratio less than 5), we used model averaging ([43,45]; function *‘*model.avg’ [42] in model parameter estimation and inference). Otherwise, we based our inferences on the single best model.

## 3. Results

**Figure 2.**
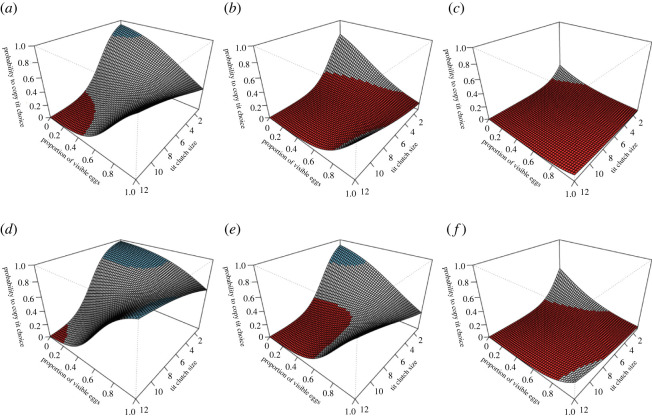
Regression surfaces (i.e. model predictions) illustrating the flycatcher probability to copy tit choice (vertical axis) in relation to tit clutch size and proportion of visible eggs in the tit nest. The regression surfaces are shown separately for young (*a*–*c*) and old (*d*–*f*) flycatcher females with minimum (*a*,*d*), mean (*b*,*e*) and maximum (*c*,*f*) tarsus length to demonstrate the main effects of flycatcher age and body size on predictions (table 1; note that the main effects only affect the elevation of the regression surface in the scale of the linear predictor). The regression surfaces are derived from a generalized linear model (table 1) using data that include choices made during egg-laying or incubation of great tits. Blue and red colour show those parts of the fitted regression surfaces where flycatchers copied and rejected great tit symbol size choices, respectively (i.e. 95% confidence intervals of the regression surface did not encompass 0.5). In the grey parts of the regression surfaces, flycatcher choices did not differ from random (i.e. 95% confidence intervals encompassed 0.5).

In our multi-model comparison, eight models were originally included in the 95% confidence set but, after removing more complex extensions of the best model, four models were retained, all of which were less than 6 AICc units from the best model (electronic supplementary material, table S1). The model including the main effects of flycatcher female age and tarsus length, the proportion of visible eggs in great tit nest and great tit clutch size, as well as an interaction between the latter two variables was clearly outperforming all the other models in explaining flycatcher choices (ΔAICc = 3.35 to the next-best model, evidence ratio of the Akaike weights = 5.46 between the two best models). Each of the number of eggs in the great tit nest, the proportion of visible eggs, their interaction, and the age and tarsus length of the flycatcher female explained flycatcher choices (table 1). We interpret these multi-dimensional results on the grounds of predictions, presented as regression surfaces in (figure 2).

**Table 1.** Parameter estimates of generalized linear models explaining flycatcher symbol choices (probability of chosen symbol size matching that of great tits) in relation to the number and visibility of eggs in great tit nest as well as the phenotype of the flycatcher female. Link function was logistic and error distribution binomial.

parameter	estimate	s.e.^a^	*z*-value	*p*-value
intercept	25.6	10.3	2.48	0.013
proportion of visible eggs	−4.34	1.37	−3.17	0.0015
great tit clutch size	−0.745	0.231	−3.22	0.0013
flycatcher age (adult)	1.12	0.489	2.29	0.022
flycatcher tarsus length	−1.21	0.523	−2.32	0.020
proportion of visible eggs × great tit clutch size	0.936	0.266	3.52	0.00044

^a^standard error.

In general, old females had a higher tendency to copy apparent tit symbol size preference than young females (figure 2) and the probability to reject tit preference strongly and consistently increased with increasing tarsus length in both young and old females (figure 2). Both adult and yearling females with short tarsi tended to copy tit preference when the number of eggs in the tit nest was small and they were mostly hidden, whereas adults tended to copy also when there was a large number of visible eggs (figure 2). However, the females with long tarsi almost systematically rejected the simulated tit symbol size preference (figure 2).

## 4. Discussion

Our results highlight two novel findings about conceptual rule use. First, we demonstrate selective relative conceptual rule transmission in the wild between two competing species via social information use by pied flycatchers observing the apparent choices of great tits. Second, we show that the decision rule of whether to copy or reject the information source's apparent preference towards nest-site characteristic (small/large abstract symbol) is strongly affected by the extended phenotype of the information source (visibility and number of eggs) and the phenotype (tarsus length and age) of the information user. Our results suggest that the reliability of the information (visibility of eggs), apparent quality of the information source (number of eggs) and phenotype (body size) of the information user modify how animals apply selective rules. Importantly, our experimental design did not include pre-training and neither rewards nor punishments, demonstrating that animals can swiftly implement such a strategy in their decision-making process. Given that most animals are consistently interacting with con- and heterospecifics and readily use intra- and interspecific social learning mechanisms [17,46], our results imply that conceptual rule use is more common in the behavioural repertoire of animals than is currently recognized.

The importance of the ecological context of the learning task—the choice of nest-site characteristics—plausibly explains the strong preferences of flycatchers. Pied flycatchers only choose nest-sites three–four times in their lifespan and it has strong effects on fitness and is under natural selection, driven mainly by nest-predation [47], towards safer elements of characteristics of the nesting site [35,48]. In a natural context, our results suggest that birds may compare nest-site characteristics, such as the width or height of the nesting tree or bush or the size of the cavity or entrance hole, of the potential and chosen sites or perceivable fitness differences of existing nests and then adjust their own choice accordingly. Such a capability may be highly adaptive and can plausibly be applied in other contexts (foraging and predator recognition) and drive the resource use of coexisting species. In asocial and within-species contexts, honeybees have been shown to form conceptual spatial relationships (above/below) between stimuli [49], which may be highly adaptive in foraging or navigation decisions [50]. In nature, coexisting individuals can readily perceive others' behavioural patterns and performance. If observable foraging success of con- or heterospecific individuals is associated with certain targets where foraging occurs (e.g. small trees over tall trees or different parts of a tree), conceptual learning should lead to selective use of available resources.

Flycatcher's copying and rejection decisions were strongly affected by the extended phenotype (visibility and number of eggs) of the information source, the tits and its own physical phenotype (tarsus length and age). These results have two ecological foundations. First, the effects of the number and visibility of tit eggs and the age of the female flycatcher on flycatcher choice match well with Loukola *et al*. [27] who showed that old flycatcher females have an overall higher probability of copying apparent tit choices than females breeding for the first time. Both high and low numbers of visible and covered eggs, respectively, increase the probability of copying while uncertainty in egg number results in rejection [17]. Higher copying probability of old females is most likely due to their previous experience with eggs, which females that are about to breed for the first time lack [22]. Plausibly the clutch size of the tit honestly reflects the quality of the decisions or behavioural repertoire of the demonstrator tit because the clutch size of great tit females has been shown to be associated with better problem-solving skills, smaller foraging ranges and smaller investment in time in foraging than non-solver females with smaller clutches [30]. Second, young and old flycatcher females with long tarsi were more likely to reject the tit symbol size preference. This was expected as tarsus length strongly correlates with body size in birds [51], which is an important proxy affecting the resource use of individuals [36]. As flycatchers are smaller than great tits in general, larger flycatchers are more similar in size to tits. Increasing similarity in size means more ecological overlap and increasing competition between the two species and this may result in rejection of great tit choices, which may then reduce the costs of coexistence [17]. This suggests that flycatchers may take their own body size into account in their decision-making and exhibit some degree of self-awareness [52].

Whatever the reason is, *rejection* instead of random choice, in a large part of the trait space (figure 2), reveals an important and rarely considered social learning strategy of rejection. Usually, if not always, social learning studies consider copying as the only response [15,32]. However, rejection can be an adaptive strategy if the tutor seems to be performing poorly [26,53] or important information related to the perceivable choices of the tutor are not available [27], because it effectively decreases the probability of adopting harmful behaviours. We hypothesize that risk aversion is a more common social learning strategy and important for the evolution of social learning than is currently acknowledged. Indeed, in the human context, the economic prospect theory by Kahnemann & Tversky [54] predicts that humans rather avoid losses than aim for gains of a similar value. Similar behaviour has also been shown in capuchin monkeys [55,56]. Hence, emphasis on avoiding poor decisions and losses rather than making good decisions and gains may be a universal bet-hedging learning strategy in the animal kingdom that has gone unnoticed in the social learning research design agenda.

Our field experiment is the first to demonstrate selective concept learning in the wild, between two competing species via social information use by observing the choices of others that included no pre-training and neither rewards or punishments. Hence, our results are significant and substantially increase our knowledge about the occurrence and implications of concept learning in nature. Given that most animals are consistently interacting with con- and heterospecifics and readily use intra- and interspecific social learning mechanisms [17,46] in guiding their own behaviour, it is likely that learning about concepts through social information use is much more common in animals than is currently known. This implies that (i) adaptive conceptual preferences can be transmitted across individuals and species boundaries among coexisting animals by copying and (ii) acquisition of non-adaptive conceptual preferences can be avoided, which may enhance the performance of the members of local animal community. Such a capacity may enable animals to adapt to local or new conditions. The adaptive significance of interspecific information use is clear because, in most systems, the majority of coexisting individuals are heterospecifics, whose different perceptual capacities, decisions, resource use and asynchrony in breeding may reveal novel information that is unattainable from conspecifics. Indeed, in many systems, there seem to be key-species which inadvertently provide information that is particularly valuable and others have adapted or learned to perceive and use that information [57–60]. Hence, to maximize understanding and benefits of the constant information flow in multi-species communities, it is likely that concept learning exists also between demonstrator and observer belonging to different species but they have not been documented so far.

## Data Availability

Data and R scripts used in this work are provided in the electronic supplementary material [61].
